# Influence of the Inclination of the Incisal Edge of Planas Direct Tracks on Deciduous Dentition with Anterior Crossbite: Finite-Elements Study

**DOI:** 10.1055/s-0041-1735435

**Published:** 2021-11-30

**Authors:** Gabriel Ribeiro de Matos, Ruben Ribeiro Neto, Almiro José Machado Júnior, Rui Barbosa Brito Junior

**Affiliations:** 1 São Leopoldo Mandic Dental School, Campinas, São Paulo, Brasil; 2 Mechanical Engineering, EMEC, Jundiaí, Brazil; 3 Department of Otorhinolaryngology, Head and Neck Surgery, University of Campinas, Campinas, São Paulo, Brazil

**Keywords:** malocclusion, crossbite, deciduous dentition, prognathism

## Abstract

**Objective** This study aimed to evaluate the influence of three different inclinations of the incisal edge of Planas direct tracks (PDTs) on the upper deciduous incisors (15, 30, and 45 degrees) through simulations using the finite-elements method.

**Materials and Methods** A three-dimensional virtual model of tooth 51 was elaborated by using the Rhinoceros computer-aided design (CAD) software. A mesh was constructed by using the Patran software, and the evaluations were processed by using the ANSYS 15.0 software. The geometry of the elements used consisted of a triangular-base tetrahedron composed of 2,167,386 elements and 3,012,995 nodal points.

**Results** Progressive alterations of proportional intensity and distribution were seen in the areas of tension on the dentoalveolar structures, with increasing inclination of the edge of the PDT. The tractive contact tensions were seen to be concentrated in the vestibular apical thirds and palatine cervical thirds, while the compressive contact tensions were in the palatine apical thirds and vestibular cervical thirds.

**Conclusion** It was concluded that a 30-degree inclination for the PDT was most appropriate for the cases of anterior crossbite in the deciduous dentition. Nevertheless, because this was a laboratory evaluation, clinical criteria and complementary examinations for each case need to be taken into consideration in making therapeutic decisions.

## Introduction

Crossbite is one of the most frequent occlusal alterations present in malocclusion. It consists of an abnormal transversal or anteroposterior relationship between the maxillary and mandibular arches when they are at their habitual maximum intercuspation. This may involve one or more teeth 1 and it may have either a functional presentation when there is a difference in the dental arch relationship between the central relationship and the habitual maximum intercuspation, or an anatomical presentation, when there is no difference between the central relationship and the habitual maximum intercuspation in the presence of crossbite. In the cases of the anatomical presentation, asymmetry at the bone base is frequently present due to adaptive growth. 2 3 4 5

Anterior crossbite in the deciduous dentition and mixed dentition has a negative influence on facial development. It has the capacity to generate morphofunctional alterations of the maxillary and mandibular bones, thus having repercussions for the entire middle part and lower third of the face. 3 4 5 6 It may lead to exacerbation of mandibular growth and restriction of maxillary and mid-face growth. These alterations may lead to development of Angle class III dental relationships, a concave facial profile, facial asymmetry and functional adaptations with regard to mastication, phonetics, facial expression, and postural relationships of the head and neck. These may have repercussions regarding the entire posture of the body. Inadequacies of the incisor enamel, periodontal alterations, and alterations to the temporomandibular joints may also occur. 6 7 8 9 10 11

In the literature, several techniques for reversing anterior crossbite are presented. These include use of fixtures comprising inclined planes made of acrylic resin; use of metal crowns; and fixed devices with 4 × 2 facial mask composition for reverse traction with or without rapid expansion of the maxilla, use of chin cups, and reconstructions using photoactivated composite resins and Planas direct tracks (PDTs). 7 8 9 10 11 12 13 14 15 . However, there is a lack of well-designed studies aimed at validating the techniques used (Borrie, Bearn, 2011).

PDTs are among the therapeutic resources of maxillary functional orthopedics and, specifically, neuro-occlusal rehabilitation. They have been shown clinically to be an efficient method for treating malocclusion and particularly in cases of crossbite. They do not depend on collaboration from the patient and act full-time on the stomatognathic system. They can be applied to young children and present low risk because they are fixed to the deciduous teeth. In these children, morphological and functional displacements of dentofacial structures are still forming and much time remains before the end of facial growth and development. 1 2 3 4 5 6 7 8 9 10 11 12 13 14 15 16 17 18 19

In the present study, the finite-elements method was used to make simulations and extrapolations with a view to seeking to understand the behavior of materials, structures and tissues, so as to guide clinical actions in cases of use of PDTs to treat anterior crossbite in the deciduous dentition. 20 21

## Materials and Methods

This study was conducted in the finite-elements laboratory of Faculdade São Leopoldo Mandic, Campinas, São Paulo, Brazil.

The following hardware was used microcomputer with 3.5 GHz Intel Quad i7 processor, 32 gigabytes of RAM DDR3 memory, and 2 GB EVGA GTX 670 video board.

The following software was used:

- Microsoft Windows 7 and Microsoft Office Word (Microsoft Corp., Redmond, Washington 98052, United States)
- Adobe Photoshop 9.0 (Adobe Systems Inc., 345 Park Avenue, San Jose, California 95110–2704, United States)
- Rhinoceros version 4.0 SR 5 (Robert McNeel & Associates, Seattle, Washington, United States)
- Patran (MSC Software, 4675 MacArthur Court, Newport Beach, California 92660, United States)
- Ansys 15.0 (Ansys, Inc., Southpointe 2600, ANSYS Drive, Canonsburg, Pennsylvania 15317, United States)

### Preprocessing

The software used in the analysis via the finite-elements method was developed to evaluate the tensions and deformations in the model.

### Information Needed for the Calculations

- The total number of nodes or nodal points
- The total number of elements involved
- A numbering system for identifying each node and each element
- The properties of the materials, such as Young's modulus (modulus of elasticity) and Poisson's coefficient, associated with each element
- A numbering system for identifying each nodal point
- The outline conditions with constraints (restriction of degrees of freedom at specific nodes)
- The type of confined delimitation
- The evaluation on the forces present in the external nodes
- The forces acting in the model

### Definition of the Models

The models were designed virtually by means of computer-aided design (CAD) through the Rhinoceros software. The respective files were imported through the Patran modeling software in the iges format. The Patran software was used to generate a mesh and all the structures were exported in a single iges files. This was imported to the Ansys 15.0 finite-elements analysis software.

To evaluate the tensions that were developed, it was sought through the models to represent the structures as closely to the clinical reality as possible. The region represented was the maxillary deciduous upper incisors, which based on human dimensions presented in the dental literature. 22 23

### Definition of the Mesh

The finite-elements method defined the modeling of real and continuous structures to form a finite set of discrete structural elements that were connected by a finite number of points named nodes. The elements formed by the division of the original structure into elements needed to have the mechanical characteristics of the structures from which they originated. 24 Each element was represented by a mathematical matrix of collective interactions among the degrees of freedom of a set of nodes. These nodes were points in space at which the degrees of freedom and forces of a structure undergoing loading were considered. The mesh of finite elements was the set of nodes and elements that represented a given structure.

**Fig. 1:**
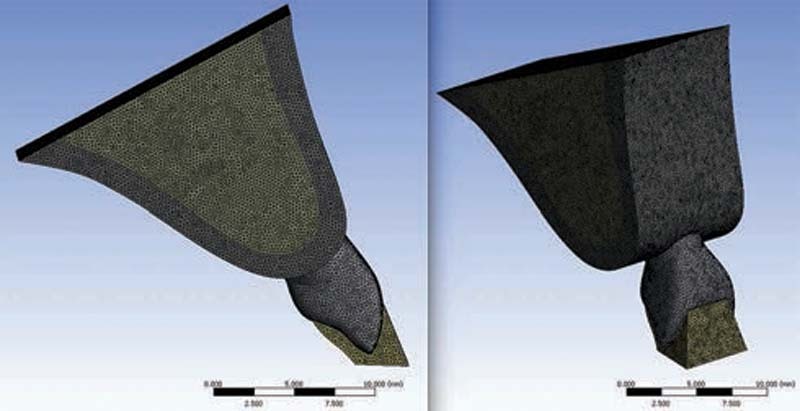
Dental and support structures made discrete and recognized as solid (distal and vestibular views). To perform the analyses, information on the properties of the structures involved in the analysis, such as Young's modulus and Poisson's coefficient, were supplied. The materials were considered to be isotropic and linear, elastic, and continuous.

The elements and nodes were identified through a numerical system. Their spatial localization was established through a system of coordinates that could be two or three dimensional. In the present study, triangular-base pyramids were used. In these, a total of 10 nodes were located at the midpoint of each edge and at the vertices of each pyramid. The final model comprised 2,167,386 elements and 3,012,995 nodes. Between these bodies, contact elements of “bonded” nature were introduced. These restricted the separation and sliding movements between the contact surfaces. To restrict displacement of the model, the elements forming the base were constrained and thus were considered stable, that is, they had three degrees of freedom, on the X, Y and Z axes, that is, zero displacement ( Fig. 1 ).

### Models

The model used was an upper deciduous incisor, with dimensions as described in Table 1 . Construction of an anterior direct track on a clinical crown was simulated. Three inclinations of the incisor edge were simulated: 15, 30, and 45 in relation to the long axis of the tooth. A vestibular-lingual and mesial-distal three-dimensional plan was investigated.

**Table 1:** Reference measurements for constructing the model (computer-aided design)

Occlusal-apical measurements of tooth 51	Occlusal-apical measurement: 16 mm Occlusal-cervical measurement: 6 mm
Vestibular-lingual cross-section	Vestibular-lingual measurement on the cervical line: 5 mm Maximum vestibular-lingual measurement of the crown: 5 mm

After the model had been imported to the Patran software, a mesh of finite elements for the different regions was generated and various properties were attributed to each structure. To perform the analyses, information available in the literature regarding the properties of the structures involves, such as Young's modulus and Poisson's coefficient, was used ( Table 2 ).

**Table 2:** Properties of the structures

Surface	Material	Modulus of elasticity (MPa)	Poisson's coefficient	Reference
Cortical bone	Cortical bone	13,700	0.30	Carter (1977)
Medullary bone	Medullary bone	1400	0.30	Carter (1977)
Resin	Z100 3M resin (ESPE)	21000	0.24	Chung (2004)
Periodontal ligament	Ligament	6.89	0.45	Fill et al (2011)
Tooth	Dentin	18600	0.31	Sano (1994) 30

These structures were considered to be continuous and the limits were constrained to avoid displacement. Each structure was considered together with its specific properties to approximate the real behavior.

### Definition of the Properties of the Periodontal Ligament

**Fig. 2:**
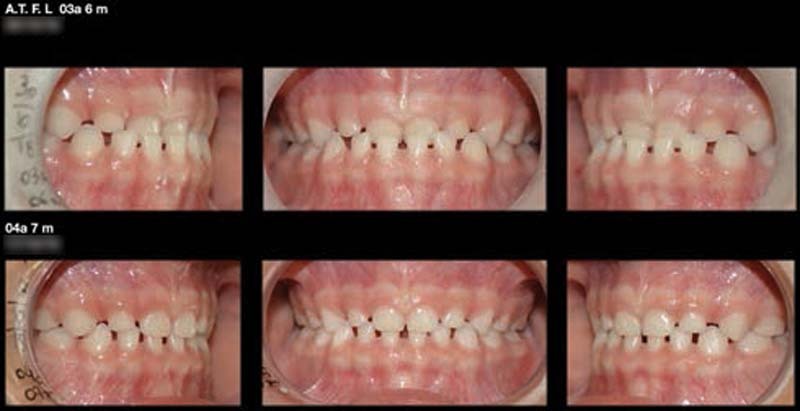
Clinical application.

An enormous variety of values were found to have been proposed in the literature for the properties of the structures that formed the periodontal ligament. The variation was up to six orders of magnitude. In this light, different values for the modulus of elasticity and Poisson's coefficient of the periodontal ligament were simulated in the software (Ansys 15.0). This was done until the behavior of the trial was close to clinical behavior, that is, with displacement within the limits of the thickness of the periodontal ligament (mean = 0.2 mm) at the time of applying the loading of the modulus of elasticity (6.89 MPa) and Poisson's coefficient (0.45) ( Fig. 2 ). Another factor that considered was the sensitivity of the periodontal ligament to the type of loading used. In this trial, the loading corresponded to the mean of the occlusal forces found in children in the anterior region of the deciduous dentition (50 N), with intermittent characteristics. This differed from the loadings used in orthodontics, which are of continuous nature and are mild forces. It should be noted that periodontal tissue responds rigidly when subjected to rapid deformation (such as mastication), but deforms elastoplastically when subjected to low-level continuous forces like those of orthodontic movement.

### Load Application

**Fig. 3:**
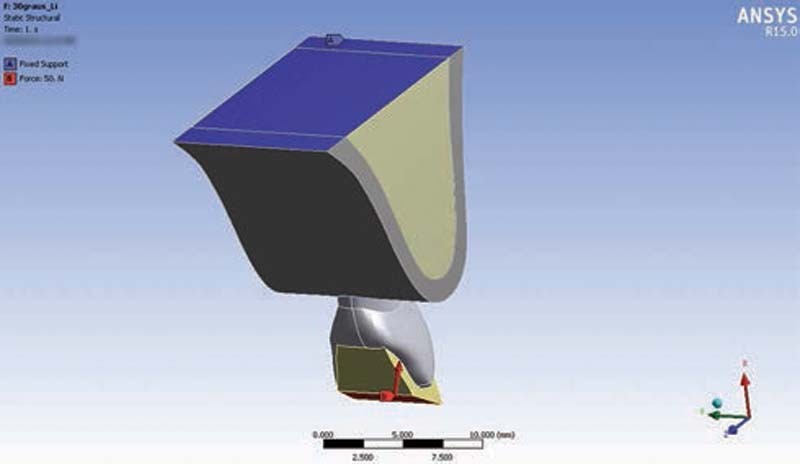
Loading of the surface of the Planas direct track (distal view).

A load of 50 N was applied to the model in a direction normal to the surfaces of the simulated incisal edge, as described in the study by Gary et al (2011) and illustrated in Fig. 3 .

### Processing

Processing was done by using the ANSYS 15.0 software, with calculation of the equations that described the model under analysis.

### Postprocessing

The results were presented as tension/deformation diagrams showing the tension distribution and numerical values. The evaluations were done through qualitative and quantitative analyses. The distribution of the colored areas showed the qualitative analysis, with the areas of concentration of the tensions. The quantitative analysis was expressed in MPa in the legend, in which the locations of the tensions with their respective numerical values and color gradients were expressed.

For the evaluations on normal stress in the model representing the structural deformations of the tooth itself, new X, Y, and Z axes relating directly to the tooth were defined.

## Results

Through processing and evaluation of the data obtained, the locations of the tractive and compressive tensions of the contact pressures were shown. This finding was defined as the most appropriate means of assessment and the one of greatest interest for describing the interrelations of the loadings in the simulated models and the bone remodeling processes relating to tooth movements.

In all the simulations analyzed, it was found that the tensions were distributed around the dental structure, bone tissue, periodontal ligament, and PDTs. They presented greatest concentration in the cervical and apical radicular thirds.

It was observed that in the cervical vestibular third, the tensions at all inclinations of the PDTs simulated were compressive. On the other hand, in the cervical palatine third, the tensions were predominantly tractive. In an inverse manner, it was seen that in the apical third, the vestibular portion showed tractive tensions and the palatine surface showed compressive tensions.

**Fig. 4:**
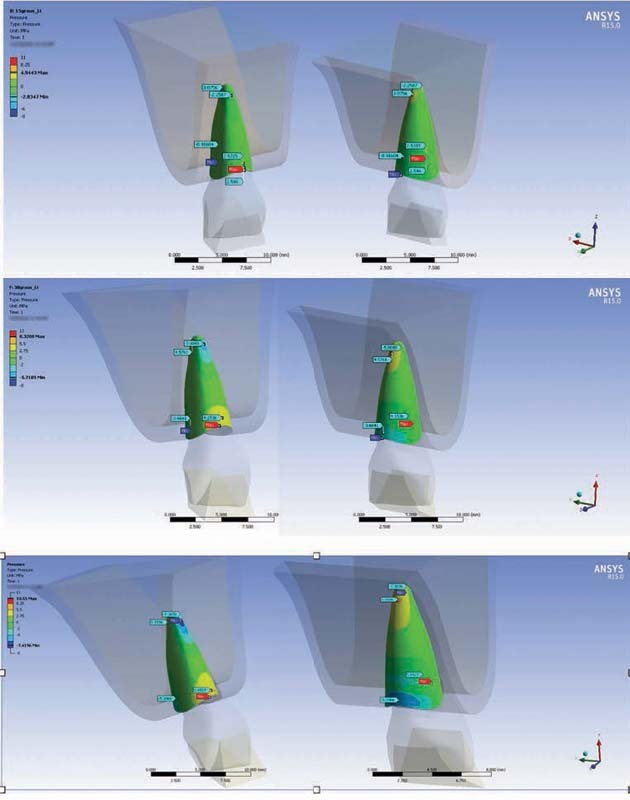
Contact pressure in vestibular and palatine views. Note the progressive increases in the intensity and area of tractive and compressive contact tensions caused by loading relating to the inclinations of the incisal edges at 15, 30, and 45 degrees, respectively.

**Fig. 5:**
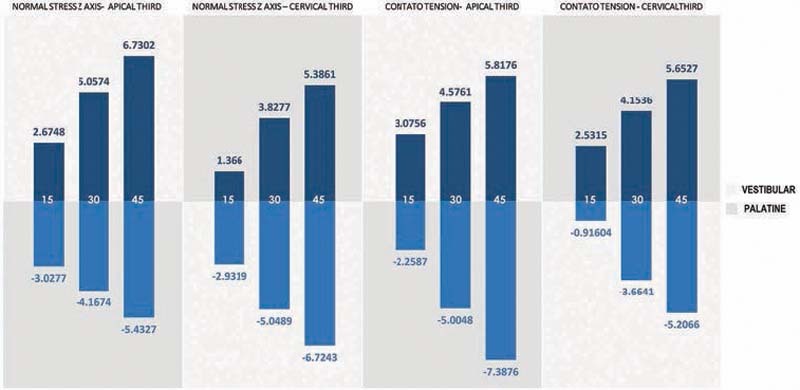
Normal stress and contact tension.

In addition, there were proportional and progressive increases, both in the intensity of tensions and in the areas of concentration of these tensions on the root surfaces, with increasing inclination of the edges of the PDTs ( Figs. 4 and 5 ; Table 3 ).

**Table 3:** Contact tension versus inclination of the incisal edge of the Planas direct tracks

Inclination of the edge of the PDT	Cervical vestibular third	Cervical palatine third	Apical vestibular third	Apical palatine third
15 degrees	2.5315	–0.91604	–2.2587	3.0756
30 degrees	4.1536	–3.6641	–5.0048	4.5761
45 degrees	5.6527	–5.2066	–7.3876	5.8176

Abbreviation: PDA, Planas direct track.

Note: Negative values indicate areas of tractive contact tensions and positive values indicate areas of compressive contact tensions (values in MPa).

**Fig. 6:**
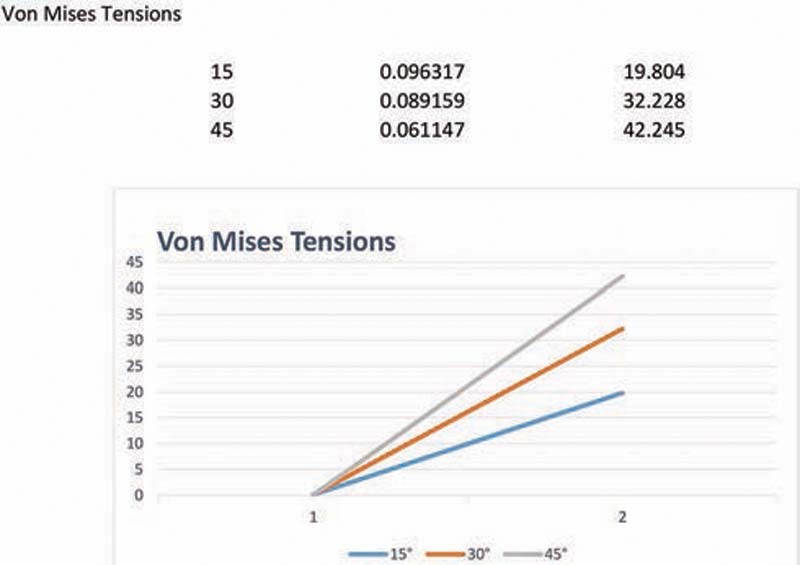
Von Mises tensions.

Regarding the distribution of Von Mises tensions, direct elevation of tension levels was observed with increasing inclination of the incisal edge of the PDTs. There was also a proportional increase in the distribution of the areas of tension on the vestibular and palatine root surfaces. The areas with elevated values were concentrated in the middle third of the root, both on the palatine and on the vestibular face. These were the locations of the peak tensions at all three inclinations simulated ( Fig. 6 ).

**Fig. 7:**
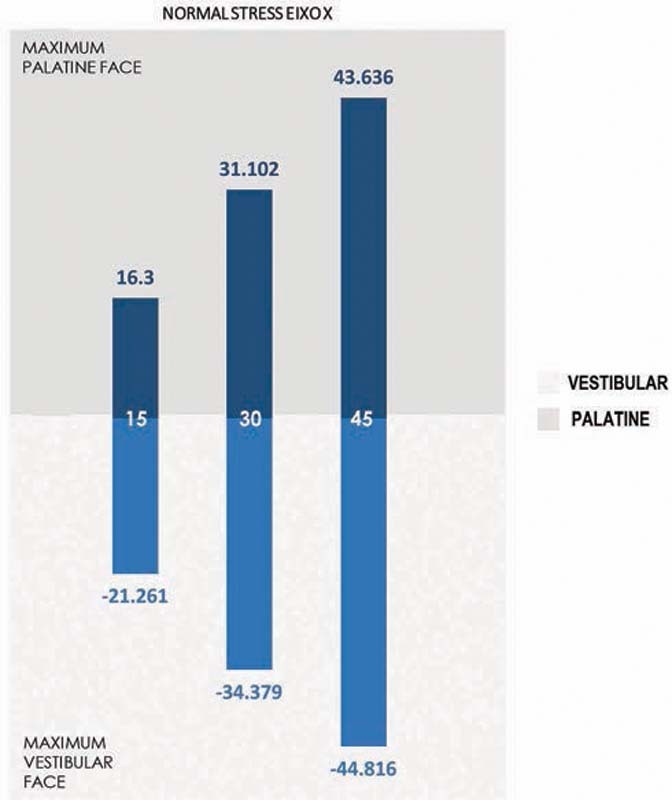
Normal stress eixo X.

In relation to the structural stress promoted by loading in the different trials (normal stress), progressive structural deformation that was directly proportional to the increase in inclination of the edge of the PDT was observed, both on the X axis and on the Z axis. There was a tendency for tooth movement vertically and horizontally toward rotating the crown in the vestibular direction, and the apical portion in the palatine direction ( Figs. 5 and 7 ).

## Discussion

Many therapeutic options are presented in the literature regarding treatments for anterior crossbite, and PDTs are among these. 1 2 3 4 5 6 7 8 9 10 11 12 13 14 15 16 17 18 19 20 21 22 23 24 25 26 27 28 The present study was conceptualized in the light of the relevance of these previous descriptions, the high prevalence of anterior crossbite, the importance of making timely differential diagnoses for these malocclusions, and their repercussions on facial growth and development processes. Through the finite-elements method, it was sought to understand the biomechanical phenomena that resulted from application of PDTs to a deciduous incisor at three different inclinations of the incisal edge (15, 30, and 45 degrees), which was then subjected to loading of 50 N. Identification of the areas where tractive tensions arose could be correlated with areas of bone neoformation, while areas of compressive tensions were corelated with bone resorption. Attention was also directed toward the capacity for tooth inclination and its relationship with the presence of the germ for the permanent incisor in the apical palatine region. Generation of an accentuated dental inclination would have the potential to influence the corridor for dental eruption of the permanent successor.

The experiments followed criteria that had already been used and described in the literature. However, they were made more sensitive by using a greater number of finite elements per area, that is, 3,012,995 nodes and 2,167,386 elements. This enabled very precise reading of the phenomena that occurred during the simulations. The results from this study corroborate the data in the literature: the finite-elements method used in the present study had previously been considered to be efficient for reading biomechanical processes. 20 21 22 23 24 25 26 27 28 29 30 31 However, enormous variation in the reported properties of the structures that form the periodontal ligament were found in the literature, varying by up to six orders of magnitude. For the present experiment, the values that fitted best were a modulus of elasticity of 6.89 MPa and Poisson's coefficient of 0.45. With these values, the finite-elements trial produced behavior close to the expected clinical behavior. The loadings for the trials were of the order of 50 N, but the duration of application was short. Thus, the load applied was intermittent and similar to the mean values for mastication in the region of the deciduous incisors. 24

With increasing inclination of the resins, progressive increases in the intensity and area of the tractive and compressive forces. This showed the importance of this treatment with regard to repercussions on the dental structures, bone tissue, and periodontal ligament. Thus, it was possible to infer a directly proportional relationship between increasing inclination of the PDTs and the intensity of the vectors that have the capacity to promote dental inclination, with the possibility of progressive apical-palatine and coronal-vestibular displacement. Because of the presence of the germ of the permanent incisor, this needs to be taken into consideration when using PDTs. On the other hand, in choosing therapy via PDTs, stabilization of the mandible during closure movements needs to be considered, so as to avoid anterior mandibular displacement that could lead to recurrence of anterior crossbite.

As this is an initial study of a broader research project, it started with laboratory tests in Finite Elements. Through extrapolation of the results found, a clinical study was started in a more oriented way and with greater predictability and safety for the treatment of MCAs in primary dentition using PDPs. Many articles related to the treatment of anterior crossbite were identified in the relevant literature, with a greater number of case reports with different therapeutic proposals. When considering the technical aspects of the construction of PDTs, these were not fully specified regarding their geometry and construction method. Due to the relevance of these aspects, we sought to measure the adequate construction of the incisal edge of PDPs aiming at jaw stability, generation of adequate functional stimuli, reduction of adverse effects to permanent successors, and reduction of recurrences of MCAs. However, this laboratory study has limitations and new studies, including clinical ones, should be performed to cover this issue.

## Conclusion

It was shown that increasing the inclination of the PDTs promoted a progressive increase in the intensity of the tractive and compressive tensions, and it also increased the distribution of these tensions across the dental structures, bone tissue, and periodontal ligament.

The compressive contact tensions on the periodontal ligament were located in the cervical vestibular and apical palatine thirds and the tractive tensions in the cervical palatine and apical vestibular thirds. These findings were compatible with the expected dental movement of the crown in the vestibular direction and the apical region in the palatine direction.

The progressive increase in inclination of the PDTs, and in particular the inclination at 45 degrees, was able to significantly move the apical region in the palatine direction and the crown in the vestibular direction. This gave rise to the potential to influence the corridor of eruption of the permanent incisor. Conversely, the inclination at 15 degrees generated the lowest horizontal vectors. However, horizontal vectors are needed for reversing the crossbite and stabilization of the mandible on the edges of the PDTs.

The inclination at 30 degrees for the edges of the PDTs presented intermediate values for apical movement in the palatine direction and coronal movement in the vestibular direction. This inclination also promoted suitable conditions for stabilization of the mandible when in contact with the PDTs. Thus, this inclination presented lower risk of interference in the evolution of the permanent successor while maintaining the capacity for reversing the crossbite. Hence, it seemed that this was the most appropriate inclination for use in cases of anterior crossbite in the deciduous dentition.

The finite-elements method was shown to have the capacity to demonstrate the repercussions of the different inclinations of the PDTs on the structures involved in the trials. Nonetheless, further studies need to be designed to deepen the knowledge of this therapeutic method. This will generate scientific evidence of greater robustness, so as to be able to apply PDTs in cases of anterior crossbite within scientific standards and with a minimum of risk for patients.
